# Aquaporin-4 in Stroke and Brain Edema—Friend or Foe?

**DOI:** 10.3390/ijms26178178

**Published:** 2025-08-23

**Authors:** Cecilia Alejandra García Ríos, Jose E. Leon-Rojas

**Affiliations:** 1 Carrera de Medicina, Facultad Ciencias de la Salud, Universidad Nacional de Chimborazo (UNACH), Riobamba 060108, Ecuador; cecygar20@hotmail.com; 2 Cerebro, Emoción y Conducta (CEC) Research Group, Escuela de Medicina, Universidad de las Américas (UDLA), Quito 170124, Ecuador

**Keywords:** Aquaporin-4, stroke, brain edema, cytotoxic edema, vasogenic edema

## Abstract

Stroke is a leading global cause of mortality and long-term disability, with cerebral edema constituting a major contributor to early neurological deterioration and poor outcomes. Aquaporin-4 (AQP4), the predominant water channel in the central nervous system, plays a paradoxical role in stroke-related brain edema, facilitating both the formation and clearance of excess fluid depending on the pathological context. This review explores the biphasic function of AQP4 across cytotoxic and vasogenic edema, emphasizing its dynamic regulation, subcellular localization, and implications for therapeutic intervention. Evidence from rodent models shows that AQP4 exacerbates cytotoxic edema in acute ischemia by promoting intracellular water influx into astrocytes, whereas in vasogenic edema, it supports fluid reabsorption and glymphatic clearance, thereby alleviating brain swelling. Human studies corroborate AQP4 upregulation in infarcted regions and suggest a potential role for AQP4 polymorphisms and circulating levels as biomarkers of stroke severity and outcome, although larger cohorts and more robust methodological designs are needed. This review also discusses emerging pharmacological strategies to modulate AQP4 activity, including inhibitors, trafficking modulators, and gene-targeted delivery systems, while highlighting challenges in achieving phase-specific modulation. Given its central role in both injury and recovery, AQP4 emerges as a promising yet complex therapeutic target for personalized management of stroke-induced brain edema. Future directions include real-time imaging of AQP4 function, genotype-stratified clinical trials, and integration of AQP4 modulation with current stroke treatment protocols.

## 1. Introduction

Stroke remains a leading cause of death and disability worldwide. Each year, an estimated 15 million people suffer a stroke, of whom ~5 million die and another 5 million are left permanently disabled [1,2]. A major contributor to early mortality in severe strokes is cerebral edema, the swelling of brain tissue due to excess water accumulation [3]. Ischemic stroke (from arterial occlusion) often triggers cytotoxic edema in its early phases, as energy failure leads to ionic pump collapse and intracellular water influx [3]. In later stages or in hemorrhagic stroke, vasogenic edema predominates due to blood–brain barrier (BBB) disruption and extravasation of fluid into the interstitial space [3]. Current edema management is largely supportive, including hyperosmolar therapy (mannitol or hypertonic saline), ventilation adjustments to prevent hypercapnia, and surgical decompression in severe cases [4]. These interventions, many dating to the mid-20th century, remain only partially effective, underscoring the need for targeted molecular therapies [4].

In this context, Aquaporin-4 (AQP4) has emerged as a crucial mediator of brain water homeostasis and edema [5]. AQP4 is the most abundant water channel in the central nervous system, predominantly expressed in astrocytic endfeet at the BBB and brain–CSF interfaces [5]. It allows bidirectional water flux across cell membranes and has been implicated in both the formation and resolution of brain edema [6,7]. This paradoxical “friend or foe” behavior of AQP4 in stroke pathology is the central focus of our review.

We conducted a structured literature search to identify studies relevant to AQP4’s role in stroke-related edema, focusing on potential mechanisms, AQP4 functionality, and future treatment avenues. The search was conducted in PubMed/MEDLINE, Scopus, and Web of Science, from inception to May 2025 for articles written in both English and Spanish. The search terms used were (“Aquaporin-4” OR “AQP4”) AND (“stroke” OR “brain infarct” OR “cerebrovascular disease” OR “cerebrovascular accident”) AND (“edema” OR “oedema” OR “brain edema” OR “brain swelling” OR “water homeostasis”). We included original research articles, systematic reviews, and meta-analyses of in vitro, animal, and human studies; conference abstracts, editorial comments, and letters to the editor. Studies not focusing exclusively on AQP4 were not included. In total, around 1000 articles were found from the search; after duplicate removal and filtering, 55 were selected based on relevance.

In this review, we will examine AQP4’s normal role in brain water homeostasis, then review evidence from experimental models and human studies discussing AQP4’s biphasic roles: exacerbating cytotoxic edema in acute ischemia but aiding clearance of vasogenic edema once BBB disruption occurs. We will also discuss translational insights and therapeutic attempts to modulate AQP4, highlighting both the challenges and opportunities in targeting this protein for stroke and edema treatment.

## 2. AQP4 in Healthy Brain Water Homeostasis

**Figure 1:**
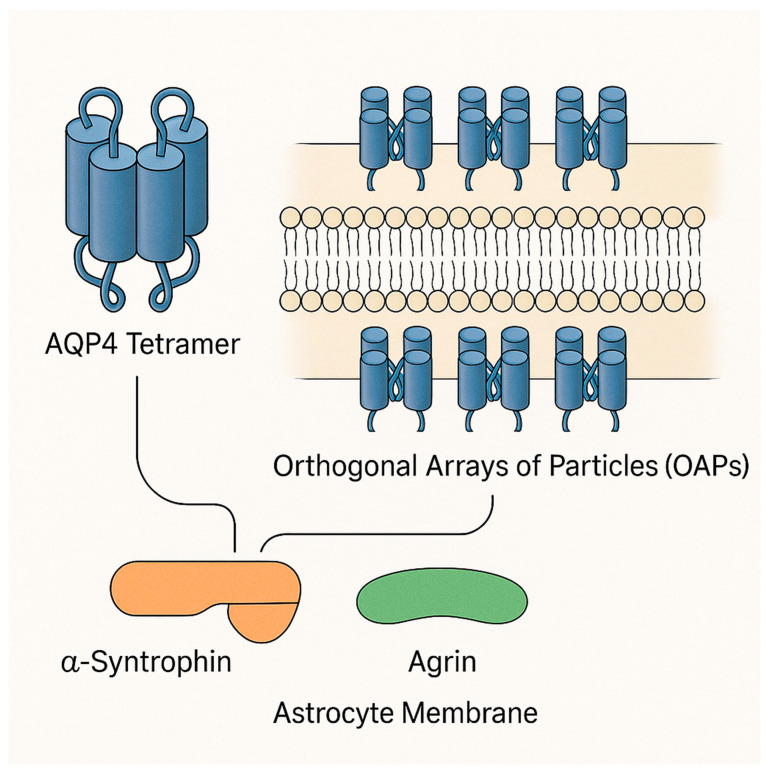
Tetrameric structure of AQP4 and the assemblies of OAPs with related anchored proteins α-syntrophin and agrin. AQP4: Aquaporin-4. Image made using Adobe Photoshop and Illustrator (Version 24.0.1).

In the healthy brain, AQP4 channels are critical for maintaining water balance and supporting neuro-glial function; it is densely localized in astrocyte foot processes that are intimately related with cerebral capillaries (the glial limiting membrane) and in ependymal lining cells of the ventricles [8]. This polarized distribution at brain–fluid interfaces positions AQP4 as a key mediator of water exchange between blood, brain interstitium, and cerebrospinal fluid (CSF) [5,8]. For example, AQP4-rich astroglial endfeet encircle microvessels, facilitating water flux that accompanies K+ uptake during neural activity (potassium spatial buffering) and volume regulation of the extracellular space [9]. Under basal conditions, AQP4 helps equilibrate osmotic gradients and prevents excessive shifts in brain water content during systemic osmolar perturbations [8,9]. Regarding the structure of AQP4, it exists as tetramers forming large supramolecular assemblies called orthogonal arrays of particles (OAPs) in the astrocyte membrane [10]. Anchoring proteins such as α-syntrophin and agrin maintain AQP4’s polarized clustering at endfeet, which is vital for its optimal water transport function [10]. The structure of AQP4 is presented in Figure 1.

**Figure 2:**
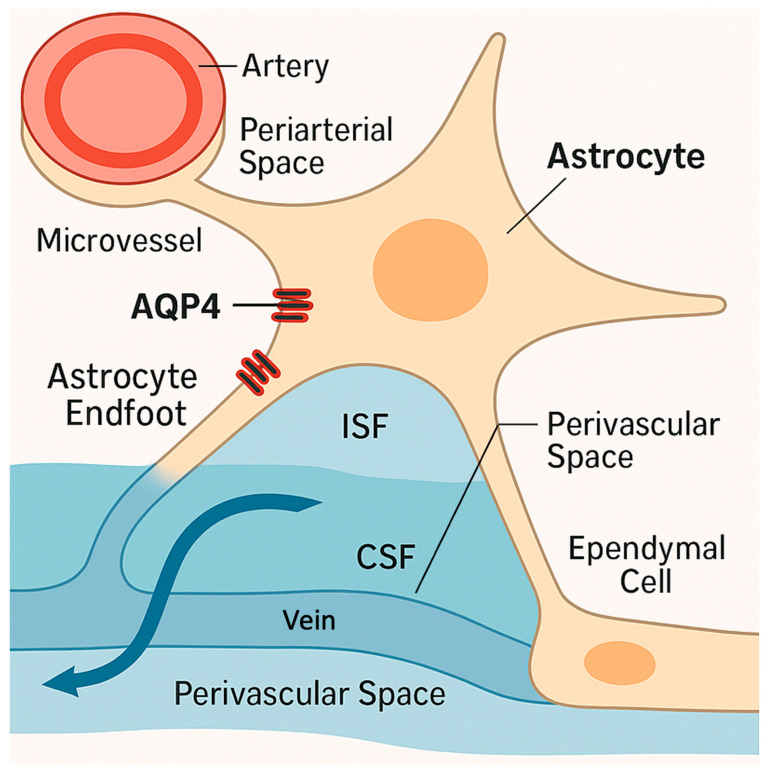
AQP4 localization and glymphatic flow in a healthy brain. AQP4: Aquaporin-4; CSF: cerebrospinal fluid; ISF: interstitial fluid. Image made using Adobe Photoshop and Illustrator.

An important aspect of AQP4’s normal role is its involvement in the brain’s glymphatic system of fluid transport and waste clearance [11]. The glymphatic system drives CSF flow through peri-arterial spaces into the interstitial compartment, facilitating convective clearance of interstitial solutes and metabolic waste via peri-venous drainage [11]. AQP4 in astrocyte endfeet greatly enhances this process by allowing rapid water exchange between the CSF and interstitial fluid (ISF) [11,12], as seen in Figure 2. In fact, AQP4 knockout animals have impaired glymphatic flow, leading to reduced CSF influx into brain parenchyma and slower clearance of ISF [12]. Conversely, high perivascular AQP4 expression is associated with efficient ISF drainage [11,12]. Thus, in the healthy state, AQP4 is a “friend”, enabling efficient water homeostasis and waste removal [13]. It helps keep brain water content in tight balance and supports neuronal microenvironment stability [13]. However, as discussed next, these same water-shuttling properties can become a double-edged sword during pathological insults like stroke.

## 3. Biphasic Roles of AQP4 in Cytotoxic vs. Vasogenic Edema

AQP4 has biphasic effects on brain edema depending on the context and timing; it can be deleterious in early cytotoxic edema but beneficial in later vasogenic edema [9]. During the acute phase of an ischemic stroke (cytotoxic edema), neurons and glia experience energy failure, leading to Na+/K+ pump malfunction and osmotic influx of water into cells [7,9]. Astrocytes, which highly express AQP4, begin to swell markedly; AQP4 accelerates this intracellular water entry, as evidenced by experiments showing that AQP4-overexpressing mice develop more rapid and severe cytotoxic edema [14]. In one study, transgenic mice with approximately 2-fold elevated astrocytic AQP4 had faster rises in intracranial pressure (ICP) and earlier herniation death after water intoxication, whereas AQP4-null mice had much less swelling [14]. The reduced rise in brain water correlated linearly with AQP4 levels, demonstrating that AQP4 is rate-limiting for cytotoxic edema formation [3,14]. Similarly, in acute focal cerebral ischemia models, AQP4 knockout mice show significantly reduced cell swelling and smaller brain water gain in the early hours, with consequently less acute tissue injury [7,15]. These findings indicate that in the initial hours of stroke, AQP4 is a “foe”, permitting water to flood into astrocytes and exacerbate cytotoxic edema and brain swelling.

By contrast, once the BBB breaks down and vasogenic edema ensues, AQP4’s abundant presence at perivascular and ependymal membranes becomes advantageous for clearing fluid [6]. In vasogenic edema, plasma leakage from vessels causes an accumulation of water in the extracellular (interstitial) space of the brain. Here, AQP4 enables efflux of edema fluid, moving water from the parenchyma into the blood or CSF, thereby reducing tissue swelling [16]. Papadopoulos et al. demonstrated this role in multiple models of vasogenic edema; in AQP4-null mice, continuous intraparenchymal fluid infusion led to dramatically higher ICP and brain water content compared to wild type (ICP ~52 vs. 26 cmH_2_O) [5,6,16]. Similarly, AQP4 knockout mice had worse outcomes and approximately 2-fold greater edema in a cortical freeze injury and in a brain tumor implantation model [5,6,16]. Deletion of AQP4 impairs fluid clearance through the glia limitans and ependymal route, resulting in refractory swelling. Consistent with this, the clinical outcome worsened in these vasogenic models when AQP4 was absent [5,6,16]. These results led to the conclusion that AQP4-mediated transcellular water movement is important for edema fluid reabsorption in vasogenic edema. In other words, in the later phase of stroke or in hemorrhagic injuries, AQP4 acts as a “friend” by facilitating the removal of excess fluid from the brain.

**Figure 3:**
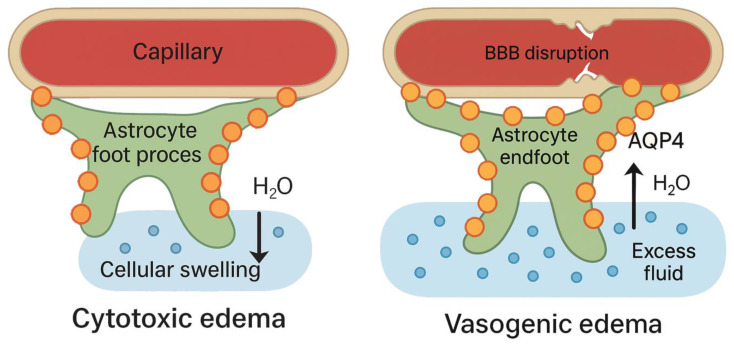
Schematic depiction of AQP4’s dual roles in stroke-related edema. Left: In cytotoxic edema (early ischemia without BBB breakdown), astrocyte foot processes (green) abundantly expressing AQP4 (orange circles) facilitate water influx from capillaries into cells, causing cellular swelling. Right: In vasogenic edema (late-stage ischemia or hemorrhage with BBB disruption), a second peak of perivascular AQP4 helps transport excess fluid (H_2_O) out of the extracellular space, aiding edema resolution. AQP4: Aquaporin-4. Image made using Adobe Photoshop and Illustrator.

The transition from foe to friend may correspond to a biphasic regulation of AQP4 expression and subcellular localization [17]. Transient ischemia studies in rodents have shown bimodal AQP4 upregulation with an early increase in AQP4 that contributes to cytotoxic swelling, followed by a second sustained increase around 24–48 h in peri-infarct astrocytes coinciding with vasogenic edema and edema resolution [15,17]. AQP4 redistribution also occurs in early stroke; AQP4 depolarizes (spreads) across astrocyte processes, while, later, it may repolarize at the endfeet to enhance fluid clearance [18]. This duality is shown in Figure 3. Consequently, an ideal therapeutic strategy might involve AQP4 inhibition during the initial hours of stroke to mitigate cytotoxic edema, but AQP4 activation or restoration in later stages hastens vasogenic edema resolution [19]. This complexity underlies the challenge of categorizing AQP4 as purely friend or foe as it is context dependent.

## 4. Evidence from Animal Models of Stroke and Edema

Much of our understanding of AQP4’s roles comes from controlled animal studies, which have looked at AQP4 function in various stroke and brain injury models; these studies consistently support a phase-dependent dual role for AQP4. Ischemic stroke models in rodents with middle cerebral artery occlusion (MCAO) have shown that AQP4 expression is dynamically altered, and its presence or absence significantly affects outcomes [20]. Early after ischemia onset, during the acute phase characterized by cytotoxic edema, AQP4 facilitates rapid water influx into astrocytes, leading to cellular swelling. In this context, AQP4 deletion is beneficial; AQP4-null mice exhibit reduced brain water uptake and edema, translating into smaller infarct volumes and improved neurological scores compared to wild type [9]. For instance, Manley et al. reported that AQP4 knockout mice had approximately 35% less edema and infarct expansion at 24 h after focal ischemia [7]. Complementarily, pharmacological blockade of AQP4 right after stroke has shown protective effects; treating mice with the AQP4 inhibitor TGN-020 within 1 h of MCAO decreased brain swelling at 24 h and improved long-term functional recovery [19,21]. By 14 days post-stroke, TGN-020–treated animals had smaller residual infarcts, less peri-infarct astrogliosis, and better neurological scores than controls [19,21]. These findings reinforce that acute AQP4 activity drives cytotoxic edema formation in ischemia and that inhibiting AQP4 in this window can be beneficial.

Conversely, in the subacute phase, when blood–brain barrier (BBB) integrity is lost and vasogenic edema predominates, AQP4 becomes essential for fluid clearance and its deletion worsens edema; therefore, AQP4 appears to switch to a protective role. As BBB breakdown allows serum proteins and fluid to infiltrate, AQP4 helps clear this vasogenic edema. Indeed, one study noted that by 3–5 days post-stroke, AQP4-null mice actually showed more edema and worse lesion encapsulation than wild type, presumably due to impaired fluid clearance [6,16]. Consistently, restoring AQP4 polarization to astrocytic endfeet in the peri-infarct zone correlates with better edema resolution and tissue healing [22,23]. These results from ischemia models underscore AQP4’s time-dependent effects, beneficial for late edema removal despite worsening early swelling. In contrast, when looking at intracerebral hemorrhage (ICH) models, hemorrhagic stroke provides a somewhat different scenario, as edema from the outset is largely vasogenic (blood leakage and plasma infiltration) [24]. Studies in a mouse ICH model (induced by intrastriatal blood injection) demonstrated that AQP4 is strongly upregulated around the hematoma and appears protective [25,26]. Chu et al. found that AQP4 knockout mice had significantly more severe edema (approximately 30% greater water content in the peri-hematoma region) and worse neurological deficits after ICH than wild-type mice; AQP4 deficiency also led to greater BBB disruption (more Evans Blue leakage) and increased neuronal death in the hemorrhagic brain [25]. The authors concluded that AQP4 deletion increases ICH-induced damage, whereas the normal upregulation of AQP4 may aid in clearing blood-derived fluid and limiting injury. Thus, in ICH (a prototypical vasogenic edema scenario), AQP4 functions as a friend, helping to remove extravasated water and ameliorate edema. Similarly, in subarachnoid hemorrhage (SAH) models, AQP4 appears beneficial; following SAH, rats show impaired CSF-ISF fluid exchange (glymphatic clearance), and this is exacerbated in AQP4 knockout rats, leading to worse early brain injury [27]. Conversely, higher perivascular AQP4 expression after SAH is associated with better glymphatic flow and reduced edema burden [28]. These hemorrhagic models reinforce that AQP4 facilitates edema fluid clearance when blood vessels are leaking.

Beyond stroke, other CNS injury models show similar patterns of AQP4 function and involvement in brain edema. In traumatic brain injury (TBI) and brain abscess models, AQP4 deletion likewise leads to higher ICP and more prolonged edema [16,22,29]. For example, in a Staphylococcal brain abscess model, AQP4-null mice had over twice the increase in water content and markedly higher ICP than wild types [16]. In spinal cord injury, AQP4 knockout also worsens edema and tissue damage, whereas strategies that prevent AQP4 mislocalization (like inhibiting calmodulin, discussed later) can reduce swelling [30]. Taken together, animal studies consistently show that eliminating AQP4 is beneficial in pure cytotoxic edema contexts but detrimental in vasogenic edema contexts (Table 1); the challenge moving forward is translating this knowledge into therapies that can modulate AQP4 at the right time and place to improve stroke outcomes. Furthermore, another relevant limitation is that most studies use young, healthy rodents, limiting generalizability; future models should incorporate age, comorbidities, and reperfusion heterogeneity.

## 5. Molecular Regulation of AQP4: Post-Transcriptional and Signaling Pathway Control

AQP4 expression and function in the brain are tightly controlled at multiple levels beyond transcription, and these regulatory mechanisms become especially relevant during stroke and hypoxic injury. Post-transcriptional regulation by noncoding RNAs has emerged as an important modulator of AQP4 in astrocytes after stroke. For example, specific microRNAs are upregulated in ischemia and act to destabilize AQP4 mRNA or inhibit its translation, thereby dampening AQP4 protein levels; notably, miR-145 and miR-29 family microRNAs directly target the AQP4 mRNA 3′UTR, reducing its expression in ischemic astrocytes [31]. Overexpression of miR-145 or miR-29a/b in stroke models suppresses AQP4, which in turn ameliorates astrocyte swelling and injury, effects that are reversed if AQP4 is knocked out or if these microRNAs are inhibited [31]. This indicates that endogenous post-transcriptional repression of AQP4 by microRNAs is a protective response limiting cytotoxic edema.

Conversely, hypoxic stress can increase AQP4 at the transcription level via oxygen-sensitive signaling thanks to the hypoxia-inducible factor-1α (HIF-1α), a master regulator in low-oxygen conditions that stabilizes and transactivates genes that facilitate adaptation to hypoxic cellular environments [29]. Under ischemia or traumatic brain injury, HIF-1α escape from degradation leads to upregulation of AQP4 gene transcription as one of its target responses [29]. Indeed, blocking HIF-1α activity in vivo has been shown to blunt the usual post-injury rise in AQP4 and significantly reduce edema formation [29]. These findings underscore that while transcription factors like HIF-1α boost AQP4 expression in hypoxia, post-transcriptional mechanisms (e.g., microRNA-mediated mRNA suppression) act in the opposite direction to restrain AQP4, suggesting a complex balance that fine-tunes AQP4 levels during stroke and that could become potential therapeutic targets if better understood.

Once the AQP4 protein is produced, post-translational modifications and protein–protein interactions critically regulate its trafficking, membrane localization, and water channel activity. Phosphorylation of AQP4 at specific serine residues has been shown to alter both its cellular distribution and function [32,33]. In astrocytes, multiple kinase pathways are activated by ischemic or osmotic stress that converge on AQP4. One key pathway involves Mitogen-Activated Protein Kinases (MAPKs), where cerebral ischemia triggers rapid phosphorylation of ERK1/2, JNK, and p38 MAPK in astrocytes [32,33]. Inhibiting p38 MAPK attenuates the post-ischemic rise in AQP4 and can reduce astrocyte swelling and cell death, suggesting this pathway upregulates AQP4 during stroke-induced stress [33]. Another major signaling axis is the cAMP/Protein Kinase A (PKA) pathway, which tends to promote AQP4 accumulation at the cell surface [32]. Cell and slice experiments demonstrate that AQP4 translocation to the plasma membrane is PKA dependent; for instance, a hypoosmotic stimulus can cause a rapid insertion of AQP4 into the membrane in astrocytes, an effect abolished by PKA inhibition [32,34,35]. Mutation of a PKA consensus site on AQP4’s C-terminus (Ser276) prevents this swelling-induced redistribution, whereas a phospho-mimetic at this site drives membrane targeting, even when PKA is blocked [32,34]. PKA phosphorylation of AQP4, thus, appears to favor its trafficking to the membrane, increasing water permeability during acute osmotic stress. In contrast, Protein Kinase C (PKC) phosphorylation has the opposite outcome; PKC activation (for example, via astrocytic vasopressin V1a receptors during injury) phosphorylates AQP4 at Ser180 in an internal loop, triggering AQP4 internalization and removal from the plasma membrane [32,34,36]. Experimentally, PKC activators have been shown to reduce brain water content and cell swelling, correlating with a downregulation of membrane AQP4 [37]. Together, these phosphorylation events form a regulatory switch; PKA-mediated phosphorylation tends to retain or insert AQP4 in the membrane (enhancing water transport), whereas PKC-mediated phosphorylation tags AQP4 for internalization and possibly degradation (limiting water uptake) [32,34]. These diverse kinase influences highlight that AQP4 membrane abundance is dynamically controlled by multiple converging signals in the ischemic brain, and the net effect (enhanced vs. reduced surface AQP4) may depend on the balance of PKA vs. PKC/MAPK activation in different injury contexts.

Beyond phosphorylation, other post-translational modifications also regulate AQP4 in the setting of stroke. AQP4 is an N-linked glycoprotein; it contains a conserved glycosylation site in its extracellular loop, and this modification can impact AQP4’s stability and oligomerization. While the role of glycosylation in stroke is not fully elucidated, by analogy to other aquaporins, it is known that glycosylation tends to increase the stability and surface expression of water channels [38]. The glycosylated form of AQP4 may be more resistant to proteolysis and may influence the formation of AQP4’s characteristic orthogonal arrays of particles (OAPs) in astrocyte endfeet [39]. Changes in AQP4’s glycosylation state under hypoxic or inflammatory conditions could, therefore, alter how much functional channel resides at the membrane, although direct evidence in stroke models remains lacking.

On the other hand, there is clear evidence that protein–protein interactions and the ubiquitin–proteasome system modulate AQP4 localization during CNS injury. AQP4’s anchoring at the perivascular membrane is dependent on the dystrophin-associated complex (including α-syntrophin and dystrophin/utrophin proteins) [40]. Ischemic stroke can disrupt this complex via proteolytic and ubiquitin-dependent mechanisms. Notably, recent work showed that after focal cerebral ischemia, the anchoring protein dystrophin-71 (DP71) undergoes ubiquitination and degradation, leading to mislocalization (depolarization) of AQP4 away from the astrocyte endfeet [40]. Loss of polarized AQP4 aggravates edema by impairing the glymphatic clearance of fluids. Inhibiting proteasomal degradation with MG132 has been shown to preserve DP71 levels and maintain AQP4 at the endfeet, which improves fluid drainage and reduced brain edema in the post-stroke period [40]. This illustrates how ubiquitin-mediated protein turnover can acutely change AQP4’s membrane presence during stroke. AQP4 itself may also be a target of ubiquitin-ligase tagging once internalized; phosphorylated AQP4 interacting with clathrin adaptors (e.g., the AP-2 and AP-3 complexes) can be routed to endo-lysosomal pathways for degradation [32]. Such AQP4 protein degradation would serve as a mechanism to limit prolonged water influx in later stages of edema. An additional layer of regulation comes from direct binding partners like calmodulin (CaM), which links calcium signaling to AQP4 behavior [30]. Astrocytes respond to cell swelling and neurotransmitter signals with rises in intracellular Ca^2+^, and Ca^2+^-activated calmodulin can bind to the AQP4 C-terminus [30]. Recent biophysical studies revealed that calmodulin binding induces a conformational change in AQP4’s cytoplasmic tail, increasing its helical structure around Ser276 and adjacent residues [30,41]. Functionally, this CaM-AQP4 interaction is necessary for rapid AQP4 redistribution; in cultured astrocytes, blocking calmodulin with a pharmacological inhibitor (e.g., trifluoperazine) prevents the usual hypotonicity-induced AQP4 membrane translocation and thereby reduces cellular water uptake and swelling. In vivo, the same CaM inhibitor was shown to attenuate edema in a spinal cord injury model, implying that interrupting AQP4-calmodulin binding can modulate edema formation [30,41]. Mutagenesis of the calmodulin-binding domain on AQP4 confirms that when AQP4 cannot bind CaM, its rate of insertion into the plasma membrane is significantly slowed [30,41]. Calmodulin, thus, acts as a calcium-sensing regulator that promotes AQP4 channel recruitment to the membrane (likely in concert with cAMP/PKA signaling), linking excitotoxic or mechanical stimuli to changes in water transport capacity.

## 6. Therapeutic Targeting of AQP4: Opportunities and Hurdles

Translating animal findings into human stroke pathology has been challenging, but emerging evidence suggests AQP4 is indeed a significant player in human brain edema. Post-mortem and tissue studies have confirmed that AQP4 is upregulated in edematous, infarcted brain regions [42,43]. In autopsied human stroke brains, AQP4 immunoreactivity has been shown to be increased in astrocytes at the peri-infarct border (edema margin) compared to normal tissue or the infarct core [42]. Notably, in these regions, AQP4 labeling extends throughout astrocyte processes (not just endfeet) and even along the subpial and subependymal surfaces [42]. This suggests a loss of AQP4 polarization and an overall increase in water channel abundance around the lesion. Aoki et al. reported that such accumulation of AQP4 at infarct edges likely reflects its role in edema development, mediating water transport from capillaries into the brain and also out via pial and ependymal surfaces [42]. Another study looking at AQP4 expression in grey and white matter of autopsied human stroke brain and controls found that AQP4 expression and lobe differ markedly between brain regions after ischemia [43]. Astrocytes in subcortical white matter show a robust upregulation and perivascular clustering of AQP4 (with a 2.2- to 6.2-fold increase), coinciding with significantly greater tissue swelling than in cortical grey matter [43]. In contrast, cortical astrocytes exhibit reduced AQP4 polarity, a change that may limit local edema formation [43]. These regional differences suggest that AQP4 abundance and distribution critically shape the extent of cytotoxic edema following stroke. In other words, human infarcts seem to recapitulate the animal findings; AQP4 is heavily mobilized in astrocytes where edema is forming, potentially contributing to swelling initially but also positioning to help drain fluid later. In patients with malignant (large) strokes, imaging studies show edema spreading in peri-infarct white matter where astrocytic AQP4 is abundantly expressed, implicating AQP4 in the rapid water uptake that can cause life-threatening brain herniation [44].

Natural genetic variation in the AQP4 gene may influence stroke edema severity in humans; a clinical genetics study by Kleffner et al. genotyped stroke patients and found a significant association between an AQP4 polymorphism and the development of severe post-stroke brain edema [45]. Specifically, a single-nucleotide polymorphism (SNP rs9951307) in the AQP4 gene was linked to lower edema risk; patients carrying the G-allele had reduced odds of massive edema formation, with an OR of 0.10 (95%CI, 0.02–0.49; *p* = 0.01) [45]. This suggests that differences in AQP4 expression or function (determined by genotype) might alter how individuals’ brains handle edema after stroke. Though this 2008 study was exploratory (41 patients), it provides proof of concept that AQP4 is a determinant of edema in humans, and it warrants further investigation in larger cohorts [45]. Another study in 363 TBI patients likewise examined AQP4 tag-SNPs and found that certain AQP4 SNPs, including rs3763043 and rs3875089, were significantly associated with 6-month functional outcomes, though not with initial injury severity or hemorrhage [46]. These mixed results could suggest that AQP4 genetic variants may influence recovery trajectories. However, the functional consequences of these polymorphisms on AQP4 expression or activity remain largely unexplored. To date, no studies have systematically evaluated whether rs9951307 or other AQP4-tag SNPs influence promoter activity, mRNA transcription efficiency, mRNA stability, splicing patterns, or protein localization. Given that the rs9951307 variant is located in a non-coding region, its mechanistic effects may involve the modulation of regulatory elements such as enhancers, transcription factor binding sites, or microRNA interaction domains. Functional studies using reporter gene assays, RNA stability measurements, and allele-specific expression analyses are needed to clarify whether this and other variants impact AQP4 levels or astrocytic water permeability. Without such data, current genotype–phenotype associations should be interpreted cautiously, as they may reflect linkage disequilibrium with causal variants or be confounded by population stratification.

Since AQP4 is an astrocytic protein, one might not expect to find it in blood, but severe BBB disruption can release astroglial fragments and proteins into the circulation, transforming them into potential biomarkers [47]. Recent work suggests that serum AQP4 levels could serve as a biomarker of stroke severity and recovery. In a 2021 pilot study of acute ischemic stroke patients receiving thrombolysis, researchers measured baseline AQP4 in blood and found an inverse correlation with infarct size and NIH stroke scale scores; paradoxically, patients with higher serum AQP4 had better early neurological improvement and outcomes [48]. One interpretation is that higher detectable AQP4 in blood reflects greater astrocytic membrane shedding from an initially robust edema response that subsequently allows fluid clearance, essentially a marker that AQP4 has been activated and possibly aided in post-stroke fluid redistribution. Alternatively, this could indicate a beneficial immune response to AQP4 fragments [48]. In any case, such findings hint that measuring AQP4 or related extracellular vesicles in blood might help prognosticate edema development or recovery potential in stroke patients. Further, increased AQP4 in CSF or blood has been noted in other conditions like intracerebral hemorrhage and even Alzheimer’s disease (where AQP4 variants correlate with amyloid clearance), underscoring a growing recognition of AQP4’s potential as systemic biomarkers [49,50]; however, more robust studies are required to properly validate its use.

Finally, direct therapeutic targeting of AQP4 in human patients has not yet been realized, but insights come from related conditions. In neuromyelitis optica (NMO), patients develop autoantibodies against AQP4 that cause astrocyte loss and severe CNS edema, effectively an autoimmune knockout of AQP4 [51]. NMO lesions often feature extensive vasogenic edema and BBB disruption, illustrating the consequence of AQP4 dysfunction in humans (though confounded by immune injury) [51,52]. Treatments that remove or neutralize anti-AQP4 antibodies (such as aquaporumab or monoclonal antibodies like eculizumab) markedly improve NMO, reinforcing how critical AQP4 is to CNS homeostasis [53,54]. While NMO is distinct from stroke, it demonstrates that losing AQP4 is harmful in the context of inflammation and vasogenic edema, aligning with the notion that AQP4 is protective for edema resolution. Clinically, no AQP4 agonists/antagonists are yet approved for stroke or edema; nevertheless, the converging evidence from human pathology, genetics, and biomarker studies suggests that AQP4 is a valid and potential translational target [55]. However, careful strategies are required, potentially genotype-guided or guided by biomarker levels, to decide when inhibiting or augmenting AQP4 might benefit patients after stroke; robust longitudinal and randomized clinical trials are required to properly determine the benefit of AQP4 targeting in stroke.

## 7. Therapeutic Challenges and Opportunities Targeting AQP4

The dualistic nature of AQP4 in edema presents a therapeutic conundrum; the optimal intervention might need to both inhibit and then later enhance AQP4 function in the same patient and at two different time points [56]. Achieving such temporal control is complex, especially because traditional drug development seeks a single agent with a unidirectional effect (e.g., an inhibitor). If one, for example, were to simply give an AQP4 blocker to stroke patients, timing would be critical; if given too early, it might reduce cytotoxic edema and tissue damage, but if continued too long, it could impair later edema clearance, worsening outcomes and becoming potentially fatal. On the other hand, an AQP4 activator given in late stroke could aid fluid removal but would be harmful if given during the initial ischemic swelling. This phase-dependent requirement is a major challenge.

Another hurdle is the lack of selective, potent pharmacological modulators of AQP4, due to it being a passive water channel with a pore that is hard to block without off-target effects [57]. Many small molecules have been tested; for example, sulfonamide diuretics like acetazolamide and bumetanide were reported to reduce edema and AQP4 expression in some models, but these drugs have multiple actions (carbonic anhydrase inhibition, NKCC1 cotransporter inhibition), and later studies found that they do not directly inhibit AQP4 water permeability [58,59]. Dexamethasone can downregulate AQP4 expression (helpful in vasogenic edema from brain tumors), but steroids have broad effects and their efficacy in stroke edema is unproven [60]. RNA interference (siRNA) against AQP4 has also been used experimentally to affect astrocyte swelling but is far from clinical use; AQP4 gene silencing has been related with changes in ischemia-related genes such as GLUT1 and hexokinase [61]. One of the few selective AQP4 inhibitors known is TGN-020, which, in rodents, can penetrate the brain and block AQP4. A recent 2025 study in a collagenase-induced ICH model showed that AQP4 activation enhances glymphatic clearance and promotes hematoma resolution. Upregulation of AQP4 improved perivascular polarization, reduced iron deposition, and improved neurological outcomes, while TGN-020 inhibition or AQP4 knockout impaired these effects; these findings reinforce the role of AQP4 as a therapeutic target in hemorrhagic stroke [62]. While not yet used in humans, TGN-020 studies are promising as, as noted, acute post-stroke administration in rats led to reduced edema and improved recovery [19,21]. The issue is that any AQP4 inhibitor would need a short half-life or a means to “turn off” its effect to allow AQP4’s switch to a beneficial role later.

A novel therapeutic concept is modulating AQP4 subcellular localization rather than blocking the pore itself. During CNS injury, signaling pathways (involving calcium-calmodulin and protein kinase A) cause internal pools of AQP4 to move to the astrocyte membrane, increasing water influx capacity [30,35]. In a recent breakthrough, Kitchen et al. (2020) discovered that inhibiting calmodulin with trifluoperazine can prevent this relocalization of AQP4; in a rat spinal cord injury model, trifluoperazine treatment kept AQP4 away from the cell surface at the lesion, which dramatically reduced edema and improved neurological recovery in rat models [30]. Essentially, by stopping AQP4 from rushing to the astrocyte endfeet during acute injury, they blunted the initial swelling without permanently eliminating AQP4. This approach focused on targeting the trafficking of AQP4, opening a new therapeutic avenue; we could transiently block AQP4 surface insertion (to prevent cytotoxic edema), then remove that block to allow AQP4 to return and clear vasogenic edema [63]. The use of an FDA-approved drug (trifluoperazine) in that study is encouraging, though its antipsychotic activity and non-specificity may limit clinical adoption. Nonetheless, it validates that edema prevention via AQP4 localization control is feasible, although the path towards a final pharmacological solution is still long. Future drugs might more selectively target the AQP4-calmodulin interaction or other anchors (e.g., the AQP4-α-syntrophin binding [64]) to achieve a similar effect with fewer side effects in stroke patients. Looking forward, combination or phased therapies might be considered. For example, a soluble small-molecule AQP4 inhibitor could be administered in the hyperacute phase of stroke (first few hours) to delay astrocytic swelling, then washed out or counteracted by a second agent that promotes AQP4 activity in the subacute phase. Gene therapy is likely too slow for stroke, but nano-delivery systems might one day deliver short-acting AQP4 blockers to penumbral tissue [65]. Another opportunity is leveraging the body’s own sleep and glymphatic mechanisms; induced sleep or slow-wave oscillations post-stroke could enhance glymphatic clearance (which is AQP4-dependent) and, thus, vasogenic edema resolution [66,67]. Any AQP4-targeted treatment must also consider its potential side effects due to AQP4 being expressed in kidney collecting ducts, skeletal muscle, and ocular tissues, though mice lacking AQP4 manage fairly well aside from CNS issues. Still, long-term AQP4 inhibition could theoretically affect muscle or vision (retinal water balance), so a brain-targeted delivery or an on-off control pharmacological mechanism would be ideal to mitigate such risks.

AQP4 is an attractive but complex therapeutic target; we provide a comparative summary of potential therapeutic strategies in Table 2. The paradox of its friend-versus-foe roles means timing is everything. At present, no drug has precisely achieved this temporal modulation in humans, and translating successes like TGN-020 or trifluoperazine in rodents to clinical practice will require innovative trial design (possibly imaging-guided or biomarker-guided in stroke patients). Yet, given the huge unmet need in managing stroke edema, where current treatments are crude and often insufficient, pursuing AQP4-based therapies remains a promising and rational strategy that should be further and seriously explored.

## 8. Future Directions

AQP4 occupies a central role in the pathophysiology of stroke-related brain edema, acting as both a culprit and a potential cure depending on the injury phase, as thoroughly discussed in previous sections. Such duality underscores why one-size-fits-all treatments for edema have failed and why precisely timed interventions that account for the dynamic roles of AQP4 are needed. Certainly, future research endeavors should aim to disentangle the signaling mechanisms that govern AQP4’s expression and localization during stroke. A deeper understanding of how AQP4 is regulated (e.g., via phosphorylation, protein interactions, or transcriptional control under hypoxia) could reveal druggable targets to modulate its activity in a phase-specific manner. The emerging concept of AQP4 polarization (and depolarization) in astrocytes is particularly intriguing as therapies that preserve or restore AQP4’s polarized distribution at astrocytic endfeet might enhance the beneficial clearance functions without increasing harmful influx [23,61]. In tandem, further development and validation of advanced imaging techniques (such as ultra-high-b diffusion MRI or tracer-based MRI of glymphatic flow) might allow clinicians to monitor AQP4 function in vivo during stroke recovery [68,69,70]; these could guide when to switch a therapy from inhibitory to promotive, truly personalizing edema management.

On the translational front, the development of AQP4 modulators, whether small molecules, peptides, or biologics, should be considered a priority. Agents like TGN-020 and new-generation AQP4 inhibitors should continue to be explored in preclinical stroke models, with attention to dosing windows and combination with reperfusion therapies. Moreover, given the role of AQP4 in long-term outcomes (e.g., astroglial scar formation and post-stroke cognitive impairment via glymphatic dysfunction), modulating AQP4 might impact not just acute survival but also chronic recovery and dementia risk [71,72]. Finally, clinical studies should incorporate AQP4-related endpoints, for instance, genotyping stroke patients for AQP4 SNPs or measuring AQP4 in biofluids to further validate AQP4 as a therapeutic target and stratify patients who might benefit from AQP4-directed treatments or classical treatments. To facilitate clinical translation, future trials should stratify patients by infarct volume, edema type, and timing since stroke onset. Ideal study designs may include the following: (1) phase 1b/2 trials of AQP4 inhibitors in large-vessel occlusion strokes treated with reperfusion, with endpoints such as early midline shift, edema volume, and 90-day modified Rankin Scale; (2) genotype-stratified designs, enrolling patients with or without AQP4 SNPs linked to edema susceptibility; (3) biomarker-guided protocols, where AQP4 levels in serum or CSF determine eligibility or dosing schedules; and (4) imaging-guided approaches, incorporating dynamic contrast-enhanced MRI or glymphatic flow imaging to assess treatment response and stage the edema phase. These efforts require interdisciplinary collaboration between neurologists, radiologists, molecular biologists, and translational scientists to ensure that modulation of AQP4 becomes a viable component of personalized stroke therapy.

Finally, several limitations should be acknowledged in the interpretation of the current review. First, while we included clinical studies suggesting associations between AQP4 polymorphisms or circulating levels and stroke-related edema, many of these investigations did not rigorously adjust for potential confounders such as hypertension, diabetes mellitus, or other vascular comorbidities known to influence both edema severity and astroglial responses. The lack of stratified analyses or multivariate models in these cohorts limits the ability to infer causality or generalize the findings across patient populations with diverse risk profiles. Second, much of the mechanistic understanding of AQP4 function in stroke stems from preclinical rodent models, often employing young, healthy animals that may not fully replicate the pathophysiological heterogeneity of human stroke, especially in aged or multimorbid patients. Furthermore, rodent models may not fully replicate human stroke pathology as interspecies differences exist, including blood–brain barrier permeability dynamics and astrocyte polarity [73]. In rodents, AQP4 is highly polarized to astrocytic endfeet, forming dense orthogonal arrays, while in humans, perivascular AQP4 distribution is more heterogeneous, especially in white matter [73]. Moreover, species-specific regulatory mechanisms, including differences in anchoring protein interactions and post-translational modifications, may affect AQP4 expression and localization; these discrepancies warrant caution in translating preclinical findings and highlight the need for human-relevant models in future research. Third, the dual role of AQP4 in edema formation and clearance, while well supported experimentally, remains difficult to monitor dynamically in vivo, and current imaging or biomarker tools are insufficient to resolve its spatiotemporal regulation with high precision in humans. Moreover, no currently available therapy targets AQP4 directly in clinical settings, and most candidate interventions remain at the preclinical or early translational stage. Finally, while our review discusses several post-transcriptional and signaling regulatory mechanisms of AQP4, the therapeutic implications of these pathways remain speculative and require further validation in well-designed experimental and clinical studies. Addressing these limitations will be essential for the successful translation of AQP4-targeted strategies into effective, personalized treatments for stroke-related brain edema.

## 9. Conclusions

AQP4 exemplifies the delicate balance in neurobiology; too much or too little activity can tip the scales between injury and repair. Tackling the challenge of AQP4 in stroke will require embracing its dual nature. By doing so, and by designing clever strategies to harness AQP4’s friend side while mitigating its foe side, we may significantly improve the management of brain edema, saving lives and improving neurological outcomes for stroke and brain injury patients worldwide. Integrating preclinical insights with real-world pathophysiology remains the critical next step for successful translation.

## Figures and Tables

**Table 1:** Comparative effects of AQP4 deletion in cytotoxic vs. vasogenic edema models.

Context	Type of Edema	Effect of AQP4 Deletion	Interpretation	References
Acute Ischemic Stroke (MCAO, water intoxication)	Cytotoxic	↓ Edema, ↓ infarct volume, ↓ intracranial pressure (ICP); improved outcomes	AQP4 facilitates rapid water influx in astrocytes, exacerbating cytotoxic swelling	[7,14,15]
Cortical Freeze Injury	Vasogenic	↑ Edema, ↑ ICP; worsened outcomes	AQP4 necessary for extracellular fluid clearance; absence impairs reabsorption	[5,6,16]
Intracerebral Hemorrhage	Vasogenic	↑ Edema volume, ↑ neuronal death, ↑ BBB disruption	AQP4 supports removal of blood-derived fluid; deletion worsens injury	[25,26]
Subarachnoid Hemorrhage	Vasogenic	↓ Glymphatic flow, ↑ early brain injury, ↑ edema	AQP4 essential for CSF-ISF exchange; its loss impairs glymphatic function	[27,28]
Brain Abscess	Vasogenic	↑ ICP, ↑ water content, impaired edema resolution	AQP4 enables fluid clearance in infection-related vasogenic edema	[16]
Spinal Cord Injury	Mixed (early cytotoxic + late vasogenic)	↑ Edema, impaired recovery; trafficking blockade beneficial	Phase-specific role; targeting localization rather than full deletion is advantageous	[30]

**Table 2:** Comparative summary of therapeutic strategies targeting AQP4 in stroke-related brain edema.

Therapeutic Strategy	Mechanism of Action	Experimental Evidence	Stroke Phase	Proposed Clinical Use	References
TGN-020	Selective AQP4 inhibitor; blocks water permeability	Reduces cytotoxic edema and improves neurological outcome in MCAO rodent models	Hyperacute (≤6 h)	Inhibition during early cytotoxic edema to limit astrocytic swelling	[19,21,62]
Trifluoperazine (TFP)	Calmodulin inhibitor; prevents AQP4 translocation to astrocyte membrane	Reduces surface AQP4 localization, astrocytic swelling, and improves outcome in spinal cord injury and TBI models	Hyperacute (≤6 h)	Prevents AQP4 redistribution to astrocytic endfeet during early ischemia	[51,53]
siRNA/antisense oligonucleotides	Gene silencing of AQP4 mRNA	Decreases AQP4 expression, alters ischemia-related gene response (GLUT1, HK), reduces edema in rodent models	Hyperacute	Potential early inhibition of AQP4 expression (limited by delivery timing)	[50,55]
Acetazolamide/Bumetanide	Reported indirect AQP4 modulation; primarily diuretics	Weak or non-specific inhibition; off-target effects; limited efficacy in direct AQP4 blockade	Unclear; preclinical only	Not recommended as specific AQP4 inhibitors	[47,48]
Dexamethasone	Downregulates AQP4 expression via glucocorticoid receptor signalling	Shown to reduce AQP4 in vasogenic edema contexts (e.g., tumors, meningitis)	Subacute (24–72 h)	May support edema resolution, but unproven in stroke	[49]
Eculizumab/Aquaporumab	Monoclonal antibodies targeting AQP4 autoimmunity	Not applicable to stroke directly; supports concept that AQP4 loss is deleterious	Chronic (for autoimmune CNS edema)	Demonstrates harm of AQP4 dysfunction; indirect relevance to vasogenic edema	[42,43]

## Data Availability

All data are available in the manuscript.
